# *Madurella mycetomatis*-Induced Massive Shoulder Joint Destruction: A Management Challenge

**DOI:** 10.1371/journal.pntd.0004849

**Published:** 2016-08-04

**Authors:** Peter S. Scolding, Mustafa A. Q. Abbas, Roa Fathelrahman Omer, Ahmed Hassan Fahal

**Affiliations:** Mycetoma Research Centre, University of Khartoum, Khartoum, Sudan; University of California San Diego School of Medicine, UNITED STATES

## Case Presentation

The patient is a 40-year-old man who presented to the Mycetoma Research Centre (MRC) in Khartoum, Sudan with a painful left shoulder mass lesion and multiple sinuses discharging purulent discharge and black grains. He experienced increasing pain and loss of shoulder function over 3–4 months, alongside nausea, vomiting, and anorexia.

Fourteen years earlier, the patient recalled a laceration to the left shoulder during a traditional sword dance. This penetrating trauma may well relate to the current infection. The clinical course of the laceration infection lasted six years. He presented to his local rural medical centre six times, undergoing two surgical incisions and curettage procedures with subsequent physiotherapy. These achieved good functional restoration, but a definitive diagnosis was not achieved. The swelling recurred sometime later. At his fifth local consultation, four months before presenting to the MRC, a differential diagnosis of mycetoma was raised and he was started on oral itraconazole. Two months later he returned for his sixth consultation at his local health centre when it was noted the swelling had increased in size, accompanied with pain, discharging sinuses, and the systemic features noted above. He was admitted to the local health facility for 11 days and treated with intravenous benzyl penicillin, cefuroxime, and analgesia. Blood test at this time showed raised erythrocyte sedimentation rate (ESR) (110/hour). No surgical biopsy or cytology smears were taken from the lesion, but, following discharge, he was referred to the MRC to be seen one month later with subsequently admission to Soba University Hospital.

He had an appendicectomy one year prior to presentation, keloid scars over the xiphisternum following cautery, traditional treatment for back pain, a snake bite resulting in fixed flexion of the right fourth finger, and childhood abdominal surgery to evacuate traumatic haematomas. There was no family history of mycetoma. The patient was a manual labourer by occupation, with a wife and four children. He had health insurance that covered around a quarter of his healthcare costs thus far; the rest was paid for via savings, sale of assets, and family support.

**Fig 1:**
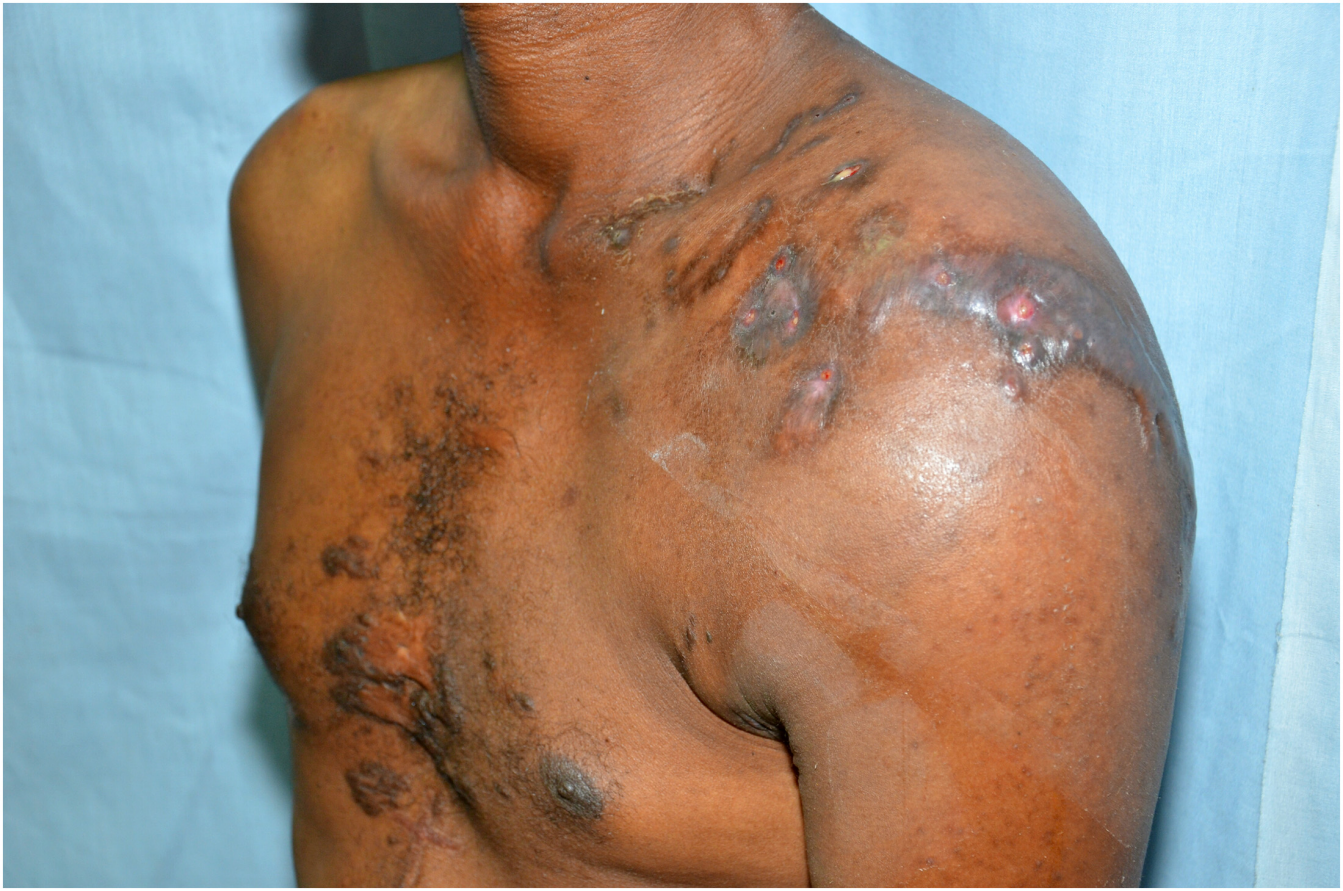
Photograph showing the mycetoma lesion around the left shoulder region.

Clinical examination revealed a mass lesion visible superficial and stretching from the lateral pectoral region to the scapula, both laterally across the deltoid region and superiorly over the middle third of the clavicle and acromioclavicular joint. It was firm with some nodules and a well-defined superficial pectoral margin but poorly defined margins elsewhere. There were 20 sinuses discharging pus with surrounding pigmentation and tenderness (Fig 1).

The shoulder joint movements were greatly impaired; he was able to abduct the shoulder to 10° with all other joint movements reduced to 0°. Tone, power, and sensations in the upper limb were intact. Further examinations showed keloids over the sternum, appendicectomy scar, and additional cautery scars over the abdomen. Cardiovascular, respiratory, and abdominal examinations were normal. No peripheral stigmata were noted.

Initial investigations highlighted normocytic anaemia of chronic inflammation (Hb 9.5 g/dl, mean corpuscular volume (MCV) of 82.1) and raised white blood count (11.6/per high power (PH), neutrophils 9.4). He was hypoalbuminemic (2.6 mg/dl [3.4–4.8]) with mild liver function abnormalities (ALP 142UI [40–129]) and bilirubin 0.5mg/dl (0.1–0.2). Alanine aminotransferase (ALT) was initially normal and rose to 68 UI (5–36) a week later. Renal profile was normal.

**Fig 2:**
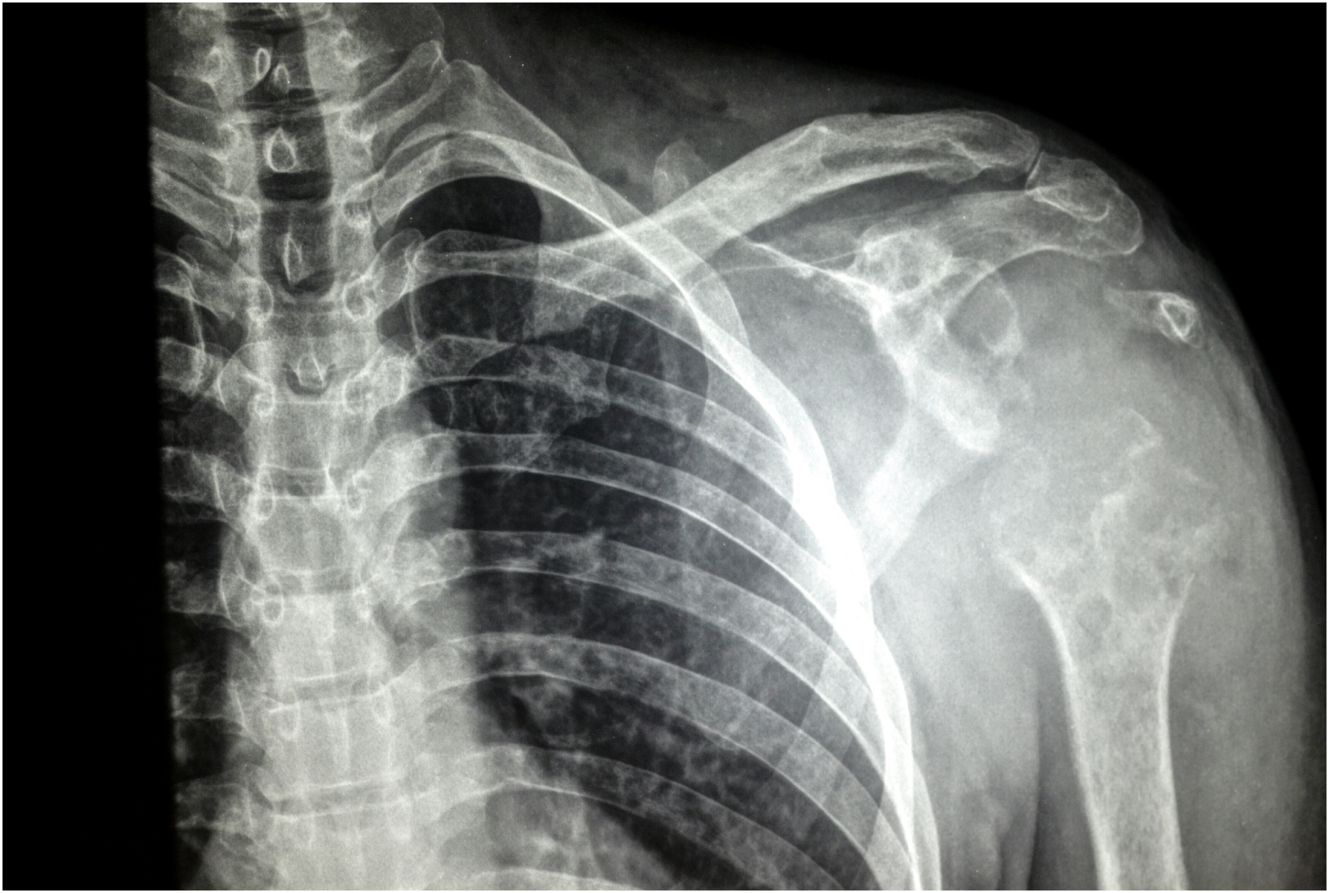
X-ray of the left shoulder joint showing soft tissue mass, complete destruction of the humeral head, and multiple cavities in glenoid cavity and coracoid process.

**Fig 3:**
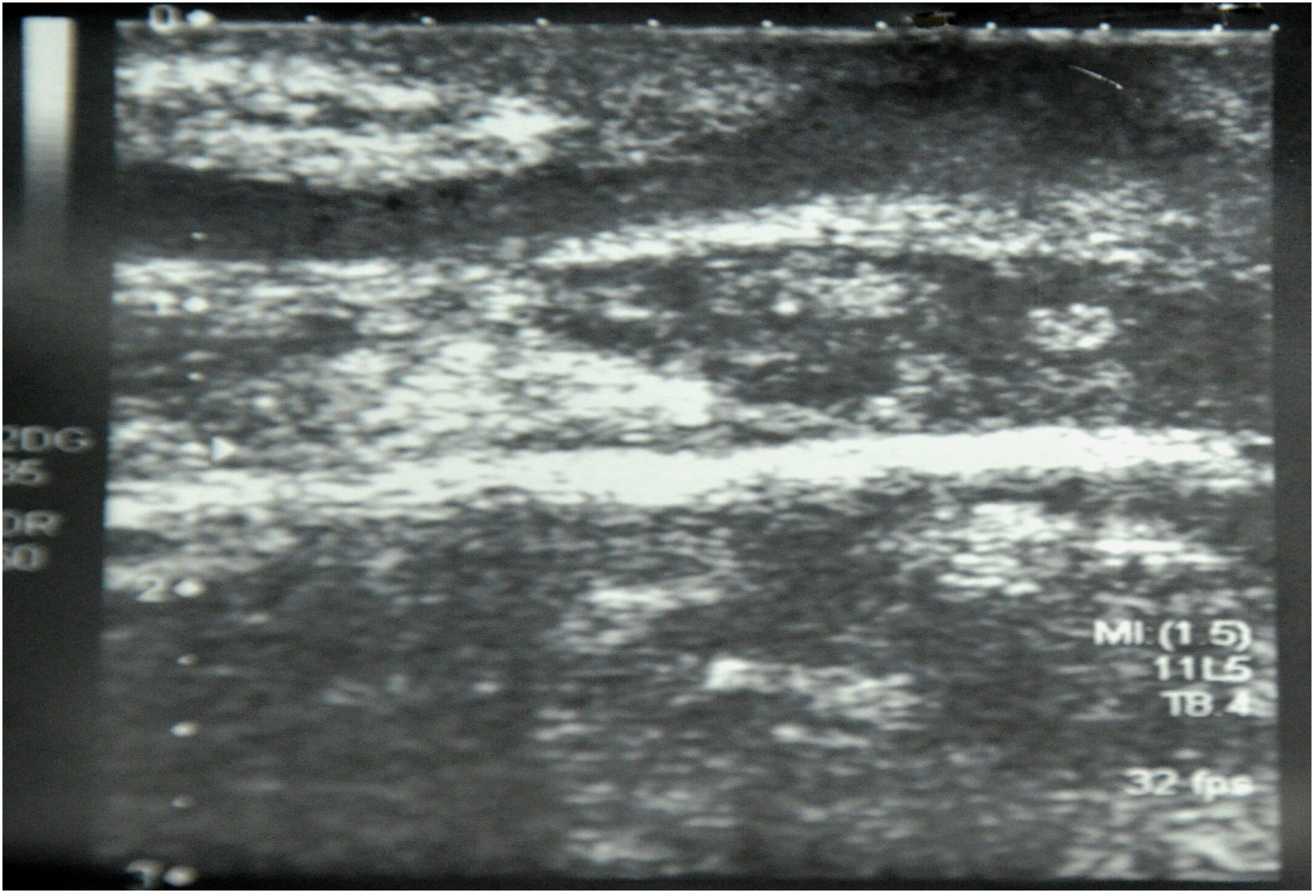
Ultrasound scan showing multiple thick-walled pockets containing multiple echogenic grains, with minimal subcutaneous oedema.

**Fig 4:**
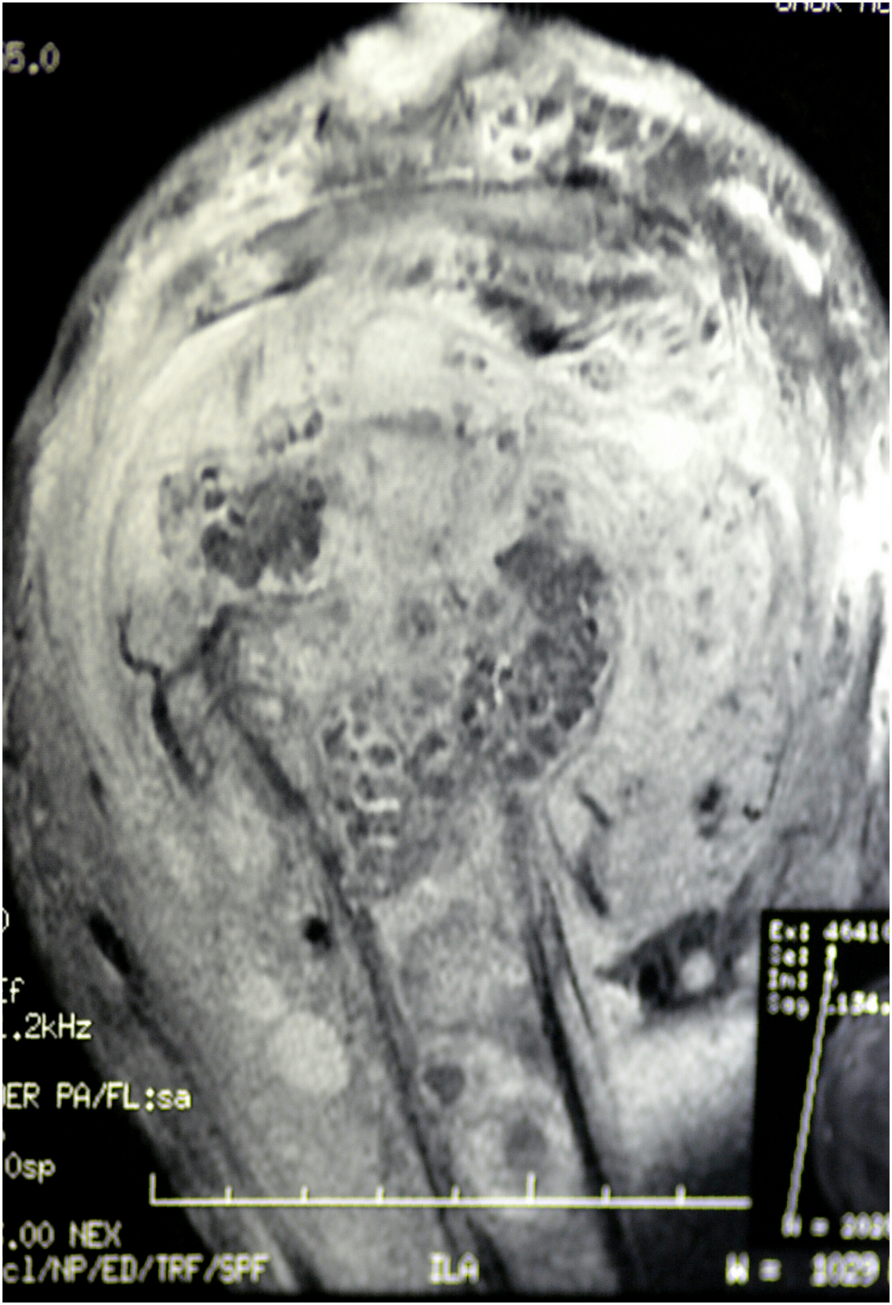
MRI showing innumerable soft tissue lesions infiltrating the rotator cuff muscles with extension into the humeral head, proximal humeral shaft, and glenoid aspect of the scapula. The acromioclavicular and glenohumeral joints were also affected, causing widening of the joint spaces. Multiple collections within affected bones and subcutaneous soft tissues consistent with abscess formation were seen. Joint effusion was noted, along with enlarged left axillary lymph nodes.

X-ray examination of the shoulder joint showed soft tissue mass, complete destruction of the humeral head, and multiple cavities in glenoid cavity and coracoid process (Fig 2). Ultrasound examination revealed multiple thick-walled pockets containing multiple echogenic grains, with minimal subcutaneous oedema (Fig 3). MRI examination showed innumerable soft tissue lesions infiltrating the rotator cuff muscles with extension into the humeral head, proximal humeral shaft, and glenoid aspect of the scapula. The acromioclavicular and glenohumeral joints were also affected, causing widening of the joint spaces. Multiple collections were seen within the affected bones and subcutaneous soft tissues consistent with abscess formation. Joint effusion was noted, along with enlarged left axillary lymph nodes (Fig 4). The differential diagnosis included eumycetoma, septic or tuberculous arthritis, and cutaneous tuberculosis.

Fine needle aspirate from the left shoulder showed *Madurella Mycetomatis* grains with numerous neutrophils, macrophages, lymphocytes, and foreign-body giant cells. Sinuses swab culture revealed no growth.

He was admitted to hospital and commenced intravenous cefuroxime on day one of admission to treat secondary bacterial infection. On day two, he received one unit of blood and commenced iron supplementation. On day three, he was restarted on oral itraconazole 400 mg. He received adequate analgesics and intravenous fluid.

By Day 20 in the hospital he felt better, vomiting stopped, appetite recovered, and he was able to eat and drink. Pain subsided at rest, though was still attendant upon any joint motion, resulting in continuing restriction to the range of movement. He will continue with medical therapy prior to surgical reassessment. It is, however, obvious that due to the extensive spread of the disease, surgical intervention would be very challenging.

## Case Discussion

Mycetoma is a unique, debilitating tropical disease causing significant morbidity across the “Mycetoma Belt” from Mexico and Brazil, across Africa (Senegal to Somalia), and from the Middle East to India [1–3]. Bacterial agents usually cause actinomycetoma, whilst fungi cause eumycetoma. Infection is believed to occur through the delivery of a causative agent into subcutaneous tissue via minor trauma [4,5]. The disease presents with a pathognomonic triad of painless subcutaneous mass, multiple sinuses, and purulent or seropurulent discharge containing grains. In advanced disease, the infection may spread to deep structures including the bones.

The case presented here is rare in the published literature. It is unique due to the site, the extensiveness of the disease process, and the involvement of multiple structures in the left shoulder joint region causing almost complete loss of the joint function. Mycetoma is usually seen in the feet and lower limbs (80%) and hands (8%); other sites including the shoulder joint are rare sites for infection [6,7]. Research in Mexico has suggested that extra-pedal cases are most commonly actinomycetoma; however, recent work in Sudan has shown that eumycetoma predominates in the extra-pedal locations [4].

In mycetoma, the external appearance is always deceiving and misleading, as seen in this patient with extensive shoulder joint destruction and severe movement limitation but with few cutaneous signs. Hence, surgery under local anaesthesia is contraindicated in mycetoma as it will result in suboptimal excision and recurrence.

A number of studies has reported on advanced presentations of mycetoma [6,7]. Indeed, patients often present late due to a range of barriers to seeking care, including scarcity of medical facilities in rural endemic areas, cost of treatment, lack of health education, and fears regarding potential amputation [4,6,7]. In the reported patient, although he was seen several times in a local health centre manned by medical assistants, neither a diagnosis was established nor was he referred to a tertiary medical facility.

The extent of mycetoma lesions can be determined by radiographic [8] and/or ultrasound imaging [9], or MRI and its grading system [10]. With the MRI Mycetoma Skin, Muscle, and Bone grading system, the reported patient’s lesions are classed as severe (10/10), with formation of sinus tract with grains in the skin and subcutaneous tissue (scores 4), formation of macro-abscess in muscle (scores 3), and bone destruction (scores 3) [10].

The treatment of this patient is a challenge due to the completely destroyed and frozen shoulder, persistent secondary bacterial infection, and the mycetoma itself. The patient needed prolonged antifungal treatment, repeated local debridement, and, most probably, arthrodesis as prosthetic joint replacement is not an option due to the chronic sepsis and mycetoma or shoulder joint disarticulation.

## Ethics Statement

A written, informed consent to publish history, findings, and images for educational purposes was obtained from the patient.

**Key Learning Points**

- Mycetoma is a badly neglected tropical disease.
- The external appearance of mycetoma is always deceiving.
- Shoulder mycetoma is rare but has serious sequelae.
- The differential diagnosis in this patient includes septic or tuberculous arthritis, cutaneous tuberculosis, or neoplasia.
- Accurate and timely diagnosis is crucial for proper treatment.
- Advanced shoulder mycetoma poses a treatment dilemma due to the extensive joint destruction, chronic sepsis, and the mycetoma itself.
